# Quantitative Metaproteomics and Activity-based Protein Profiling of Patient Fecal Microbiome Identifies Host and Microbial Serine-type Endopeptidase Activity Associated With Ulcerative Colitis

**DOI:** 10.1016/j.mcpro.2022.100197

**Published:** 2022-01-13

**Authors:** Peter S. Thuy-Boun, Ana Y. Wang, Ana Crissien-Martinez, Janice H. Xu, Sandip Chatterjee, Gregory S. Stupp, Andrew I. Su, Walter J. Coyle, Dennis W. Wolan

**Affiliations:** 1 Department of Molecular Medicine, The Scripps Research Institute, La Jolla, California, USA; 2 Scripps Green Hospital Division of Gastroenterology, La Jolla, California, USA; 3 Department of Integrative Structural and Computational Biology, The Scripps Research Institute, La Jolla, California, USA

**Keywords:** microbiome metaproteomics, ComPIL, ulcerative colitis, de novo peptide sequencing, fluorophosphonate, ABPP, activity-based protein profiling, chemoproteomics, ABPP, activity/affinity-based protein profiling, BLAST, basic local alignment search tool, CD, Crohn’s disease, ComPIL, comprehensive protein identification library, DNA, deoxyribonucleic acid, FP, fluorophosphonate, GI, gastrointestinal tract, GO, gene ontology, GWAS, genome-wide association studies, IBD, inflammatory bowel disease, LC-MS/MS, liquid chromatography tandem mass spectrometry, MAG, metagenome assembled genome, MS, mass spectrometry, NCBI, National Center for Biotechnology Information, PBS, phosphate buffered saline, PCA, principal component analysis, PPI, protein–protein interaction, PSM, peptide-spectrum match, QIIME, quantitative insights into microbial ecology, rRNA, ribosomal ribonucleic acid, STRING, search tool for recurring instances of neighboring genes, UC, ulcerative colitis

## Abstract

The gut microbiota plays an important yet incompletely understood role in the induction and propagation of ulcerative colitis (UC). Organism-level efforts to identify UC-associated microbes have revealed the importance of community structure, but less is known about the molecular effectors of disease. We performed 16S rRNA gene sequencing in parallel with label-free data-dependent LC-MS/MS proteomics to characterize the stool microbiomes of healthy (n = 8) and UC (n = 10) patients. Comparisons of taxonomic composition between techniques revealed major differences in community structure partially attributable to the additional detection of host, fungal, viral, and food peptides by metaproteomics. Differential expression analysis of metaproteomic data identified 176 significantly enriched protein groups between healthy and UC patients. Gene ontology analysis revealed several enriched functions with serine-type endopeptidase activity overrepresented in UC patients. Using a biotinylated fluorophosphonate probe and streptavidin-based enrichment, we show that serine endopeptidases are active in patient fecal samples and that additional putative serine hydrolases are detectable by this approach compared with unenriched profiling. Finally, as metaproteomic databases expand, they are expected to asymptotically approach completeness. Using ComPIL and *de novo* peptide sequencing, we estimate the size of the probable peptide space unidentified (“dark peptidome”) by our large database approach to establish a rough benchmark for database sufficiency. Despite high variability inherent in patient samples, our analysis yielded a catalog of differentially enriched proteins between healthy and UC fecal proteomes. This catalog provides a clinically relevant jumping-off point for further molecular-level studies aimed at identifying the microbial underpinnings of UC.

## Highlights

• Identified 176 significantly altered protein groups between healthy and UC patients.
• Serine-type endopeptidase activity is overrepresented in UC patients.
• Fluorophosphonate ABPP shows that endopeptidases are active in fecal samples.
• ABPP enrichment helps identify additional putative serine hydrolases in samples.
• *De novo* sequencing used to estimate number of MS2 spectra unidentified by ComPIL.

## Graphical Abstract

**Figure:**
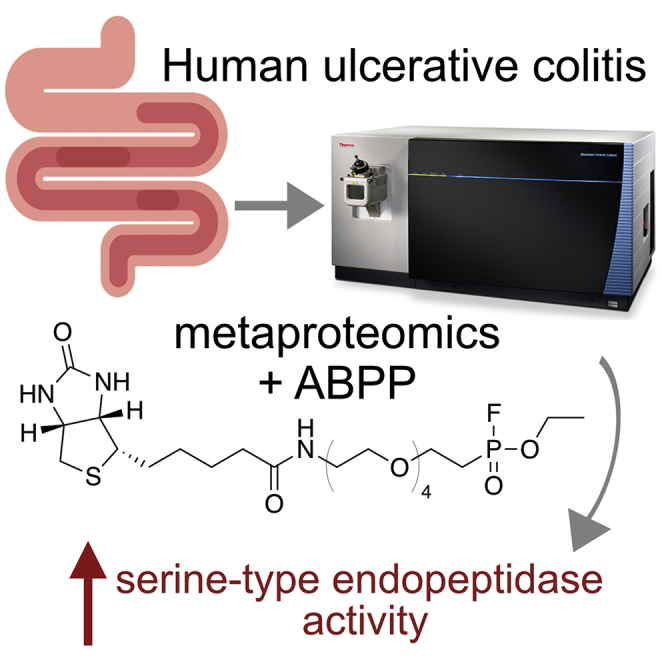

## In Brief

Thuy-Boun *et al.* quantitatively compare the stool microbiomes of healthy and ulcerative colitis patients with label-free data-dependent LC-MS/MS proteomics. Their analyses identified 176 significantly enriched protein groups between the two cohorts, and serine-type endopeptidase activity was one such functionality overrepresented in UC patients. Pre-enrichment of the clinical samples with a biotinylated fluorophosphonate probe further demonstrated that serine endopeptidases are active within the patient fecal samples and that additional putative serine hydrolases were identified by this approach compared with unenriched profiling.

Inflammatory bowel disease (IBD) is a chronic medical condition characterized by relapsing inflammation of the gastrointestinal (GI) tract. This disease is broadly divisible into two categories based on where inflammation occurs. In ulcerative colitis (UC), inflammation is restricted to the large intestine, while in Crohn’s disease (CD), inflammation can occur anywhere along the GI tract (1, 2). In addition to reduced life expectancy, IBD patients can suffer dramatic quality-of-life reductions and are at increased risk to develop gastrointestinal tract malignancy (3). The incidence and prevalence of IBD in developed countries have steadily increased in the last few decades, making this disease a public health concern with a potentially heavy cost burden due to a requirement for long-term management (4). Targeted cures for UC and CD are highly desirable, but the search for such treatments is hampered by our incomplete understanding of disease development. Genome-wide association studies (GWAS) have identified over 200 genetic loci associated with UC and CD, but the polygenic nature of these conditions explains only a minor portion of disease incidence (5, 6, 7). Concordance rates of about 30% for CD and 15% for UC among monozygotic twins suggest a significant nongenetic contribution to disease development (8). Because our gut microbes are in perpetual contact with our GI tracts, they comprise important but ill-defined environmental variables that many studies have implicated in IBD development. IBD triggers are unknown, but its progression is hypothesized to be amplified by inappropriate host–microbe interactions that lead to dysbiosis and, eventually, observable gross pathology (9, 10, 11, 12, 13).

Efforts to identify potential microbial drivers of IBD are ongoing but stymied by the immense taxonomic complexity of the gut microbiota. The gut harbors hundreds of distinct species per individual, which can change over time and with perturbations to host lifestyle or xenobiotic exposure (14, 15, 16). Campaigns to characterize and monitor the gut microbiota frequently utilize amplicon and metagenomic sequencing technologies, which can provide information about microbial community structure, genetic potential, and transcriptional activities (17, 18). As the collective size of shotgun metagenomic sequence space has expanded from these efforts, so too have the opportunities for liquid chromatography tandem mass spectrometry (LC-MS/MS)-based metaproteomics (19), which rely on protein reference databases constructed from translated genome sequences (20). Host protein profiling is a straightforward process given the relative completeness of host (*e.g.*, human, mouse, *etc.*) genome assemblies, but microbiome protein profiling is more difficult due to the high strain diversity and presence of unculturable microbes in the gut (21). Sample-matched MAGs (metagenome assembled genomes) delivering good spectrum match rates have become an effective solution but can be cost-limiting and require expertise (15, 22, 23, 24, 25). In addition, manual reference database curation has proven to be an important consideration in metaproteomics but becomes computationally burdensome as community diversity expands (26). To address this problem, we developed the Comprehensive Protein Identification Library (ComPIL), a large and scalable proteomics database generally intended for metaproteomics studies (27). In its current iteration (ComPIL 2.0), it houses >4.8 billion unique, tryptic peptides derived from >113 million bacterial, archaeal, viral, and eukaryotic parent protein sequences assembled from public sequencing repositories (28). With periodic incorporation of new sequences from shotgun metagenomics repositories, we envision that ComPIL will help enable interlaboratory consonance in the global interpretation and communication of bottom-up metaproteomics results. In addition to enabling the direct, large-scale observation of proteins in a complex mixture, LC-MS/MS-based metaproteomics techniques obviate a requirement for intact cells, facilitate the observation of posttranslational protein modifications, and enable functional interrogation of new or incompletely annotated proteins through such cognate techniques as activity- and affinity-based protein profiling (ABPP) (29, 30).

Relative to metagenomics, LC-MS/MS-based metaproteomics are less commonly applied and more rarely employed in IBD studies. In fact, the first large-scale endeavor to identify proteins from a microbial biofilm community was only disclosed by Banfield, *et al.* in 2005 (31, 32, 33). In 2009, Jansson, *et al.* leveraged high-resolution LC-MS/MS to demonstrate the viability of large-scale metaproteomics in fecal samples collected from a twin pair (34). The aforementioned study demonstrated for the first time that bottom-up LC-MS/MS-based proteomics technology is suitable for such a complex environment and that it could generate a model of the gut microbiome that is orthogonal to that produced by metagenomics. Since then, several groups have deployed bottom-up metaproteomics to investigate the etiology of IBD in humans by examining patient fecal extracts, intestinal biopsy tissue, and/or blood samples (35, 36, 37, 38, 39, 40, 41, 42, 43, 44). Additionally, several groups including our own have paired traditional proteomic profiling with ABPP techniques in microbiome samples to detect and annotate key protein functionalities often undetectable without preenrichment (45, 46, 47, 48).

The insights garnered from previous metaproteomics studies are valuable for forming a consensus about the constellation of IBD-related environmental factors. We aim to contribute to this nascent pool by presenting a combined 16S rRNA gene amplicon sequencing and metaproteomics analysis of fecal samples from healthy volunteers and ulcerative colitis patients to identify novel proteins associated with health or disease. Using a pipeline that incorporates a novel, strong-acid sample preparation procedure (49), label-free high-resolution LC-MS/MS, and the ComPIL database coupled to the ProLuCID/SEQUEST search engine (20, 50, 51), we identify 176 protein groups and several gene ontology (GO) terms enriched in either cohort (52). We show that proteomics can provide a more complete picture of gut microbiome taxonomy that includes host, microbial, and even dietary proteins. Using ABPP, we demonstrate that not only are microbiome proteins enzymatically active after collection, but additional proteomic depth can be achieved using ABPP enrichment strategies. Finally, using *de novo* peptide sequencing tools, we provide a means for estimating the size of database-elusive peptide space in our LC-MS/MS data, enabling a rough estimation of metaproteome completeness. This measure can help shape future decision-making processes regarding the need for additional shotgun metagenomic work to support a given metaproteomics study.

## Experimental Procedures

### Patient Sample Collection

Collection and use of all patient samples were approved by the Office for the Protection of Research Subjects at Scripps Research and Scripps Green Hospital (IRB protocol IRB-14-6352). The written informed consent was obtained from all subjects in accordance with the Declaration of Helsinki.

Volunteers self-collected their own stool samples using administered standardized in-home sample collection kits and were instructed to immediately freeze specimens at −20 °C. Samples were stored in provided consumer-grade −20 °C minifreezers immediately after collection, transported by courier services on dry ice, and stored in laboratory-grade freezers at −20 °C until microbial extraction. Collected stool samples were highly heterogeneous in color, texture, and viscosity both before and after microbial extraction.

### Microbe Extraction

Stool samples were thawed to room temperature, diluted in PBS (pH 7.4), vortexed thoroughly to yield slurries, and then centrifuged at 100*g* for 1 min. The flocculent upper layer was extracted, filtered over 70 μm nylon mesh cell strainers to remove large, recalcitrant masses, and then centrifuged at 8000*g* for 5 min to pellet. Pellets were rinsed twice with PBS, then resuspended in PBS to a density of 100 mg wet microbial pellet per 500 μl of suspension.

### DNA Extraction, 16S rRNA Gene Sequencing, and Data Processing

Microbial DNA was extracted from thawed fecal microbe aliquots using a fecal/soil extraction kit (Zymo Research, Irvine, CA, USA). In total, 50 to 100 ng of DNA per patient sample was submitted to the Scripps Research genomics core for next-generation sequencing, which was performed using the MiSeq platform (Illumina Inc). For taxonomy based on 16S rRNA gene amplicon sequencing, we targeted the bacterial 16S V4 region using a 300 bp paired-end approach aiming for 100,000 reads per sample. Reads were taxonomically mapped using QIIME2 (and associated plug-ins) and classifiers created from the SILVA 132 database (53, 54). For access to raw data, see Zenodo repository (55). For detailed methods, see Additional File 1.

### Protein Extraction, Protein Digestion, Proteomics Data Collection

Protein was extracted according to a previously described protocol (49). Extracted protein was resuspended in H_2_O, and concentration was measured by BCA assay (ThermoFisher). Extracted microbiome protein (100 μg) was reduced, alkylated, trypsinized, and desalted (ZipTip C18, MilliporeSigma) prior to LC-MS/MS analysis. Desalted peptides (1 μg) were separated using a 4-h C18 gradient by nano-flow liquid chromatography coupled to an Orbitrap Fusion Tribrid (Thermo Fisher) operating in data dependent mode. Both MS1 and MS2 spectra were recorded in the Orbitrap at 120K and 30K resolution, respectively. For detailed methods, see Additional File 1.

### Proteomics Data Analysis

We collected a total of 2,829,920 MS2 spectra between all 18 patient samples. These spectra were searched against the ComPIL 2.0 database (contains 4.8 billion unique tryptic peptides from >225 million forward and reverse protein sequences) (27, 28) using the ProLuCID/SEQUEST search engine (20, 50, 51). In total, 523,155 (18.5%) MS2 spectra were mapped to 54,378 distinct peptides at a 1% peptide false discovery rate (two peptide per protein minimum) using a target-decoy strategy (56). In total, 576,625 protein sequences were identified and clustered into 95,000 protein groups at a 95% sequence similarity cutoff using CD-HIT (57, 58). Quantification at the MS1 level was performed using FlashLFQ with a match-between-runs strategy enabled (10 ppm precursor tolerance, 15 min window) (59). Peptide MS1 area-under-the-curve intensities were mapped to protein groups. Intensity belonging to peptides that mapped to >1 protein group were excluded. After removing protein groups with too many missing values (protein groups were removed if: (1) both conditions contained only null values, (2) one condition contained null values and the other contained <4 non-null values, or (3) both conditions contained <4 nonnull values each), 4622 protein groups remained for differential expression analysis, which was performed using Limma as part of the DEP package in the R statistical environment (60, 61). Protein groups were annotated with GO terms using InterProScan; these annotations were used for GO enrichment analysis in the GOstats package. GO relative abundance analysis was performed before removal of protein groups with too many missing values (62, 63, 64). Peptides were mapped to their respective taxa of origin using Unipept (65, 66, 67). Note that ideally, metaproteomics should be performed in conjunction with metagenomic sequencing to generate matched customized proteome databases. Protein provenance could more confidently be traced in this scenario enabling greater precision during taxonomy analysis. In the absence of matched metagenomic data, Unipept can provide peptide-based taxonomic mapping information but may do so with less accuracy. Peptides were then mapped to taxa to construct relative abundance tables and plots. Finally, *de novo* peptide sequencing was performed in Novor (68) and database-*de novo* peptide comparisons were performed using the Scikit-bio Python library. For more detailed methods, see Additional File 1. For access to LC-MS/MS data, see PRIDE repository PXD022433 (69). For *de novo* datasets, protein fasta files, and protein group files (CD-HIT clusters), see Zenodo repository (55) 10.5281/zenodo.5717460.

### Experimental Design and Statistical Rationale

Single time-point stool samples from 18 patient volunteers were collected and analyzed. Healthy (n = 8, M/F ratio = 5:3, mean age = 44 years) and UC (n = 10, M/F ratio = 8:2, mean age = 46 years) volunteers consisted of a mixture of males and females between the ages of 21 to 76 years (global mean age = 45 years, global median age = 39 years) at the time of enrollment. UC patients presented with mild to severe symptoms and a range of Mayo scores (0–9) during enrollment. Individuals with BMI values >60, as well as any recent antibiotics usage (<3 months prior to sample collection), severe diarrheal illnesses, or *Clostridium difficile* infections were excluded.

For unenriched proteomics experiments, eight healthy biological replicates were compared against ten UC biological replicates (one technical replicate per biological replicate). Analyses did not rely on isotopic labels or internal standards. Instead, total protein and peptide concentrations were measured and normalized by BCA assay prior to LC-MS/MS. We chose to normalize samples by protein concentration to simplify comparisons, as collected patient stool samples were highly variable in volume, hydration, and consistency. This implies that in this study, proteins/protein group compositional fractions rather than absolute protein concentrations were compared between patients. ProLuCID/SEQUEST and DTASelect were used for peptide identification at a peptide-level FDR setting of 1% using a target-decoy strategy (20, 50, 51, 56, 70). Quantification was performed by MS1 peak intensity using a match-between-runs strategy. All protein sequences were grouped/clustered at a 95% sequence similarity cutoff using CD-HIT, then peptide intensities were mapped to protein groups/clusters (58). Peptides mapping to >1 group/cluster were excluded. Peptide intensities were summed within protein groups/clusters unless otherwise noted. Protein group/cluster intensities were normalized using the Limma/DEP package function “normalize_vsn” (variance stabilizing normalization) within the R statistical computing environment (60, 61). Protein group differential enrichment testing was performed using the “test_diff” function within the Limma/DEP package within the R statistical computing environment (60, 61). Differential testing *q*-values were calculated using the “qvalue” package in the R statistical environment, and a threshold of *q* < 0.1 was chosen as a relevant cutoff value for further analysis and discussion (71). Note that at more strict *q*-value thresholds, several protein groups were found to be significant (68 protein groups significant at *q* < 0.01, 55 protein groups at *q* < 0.001), but the modest *q* < 0.1 threshold was chosen to enable a more broad view of potential disease-associated protein functions with the acknowledged caveat that approximately 18 of 176 significantly enriched protein groups are false positives. Please see Additional File 1 for more details.

For FP-probe-enriched proteomics experiments, one healthy biological replicate and two UC biological replicates were analyzed by LC-MS/MS (one technical replicate for each sample). The stool samples used for these FP-enriched experiments were identical to patient stool used earlier for unenriched experiments. These FP-enriched experiments were qualitative and not statistically powered.

## Results

### Metaproteomics Yields a More Comprehensive Taxonomic Diversity Than 16S rRNA Gene Analysis

At the kingdom level, LC-MS/MS-based proteomics identified peptides mapping to bacteria (38.3%), eukaryotes (43.5%) (including host), archaea (<0.1%), and viruses as well as a significant proportion of unassigned peptide (18.2%) [Additional File 1, supplemental Fig. S1]. In contrast, a majority of 16S reads (>99%) were attributable to bacteria with a much smaller proportion attributable to archaea (<0.1%) and <0.1% remaining unassigned [Additional File 1, supplemental Fig. S1].

**Fig. 1:**
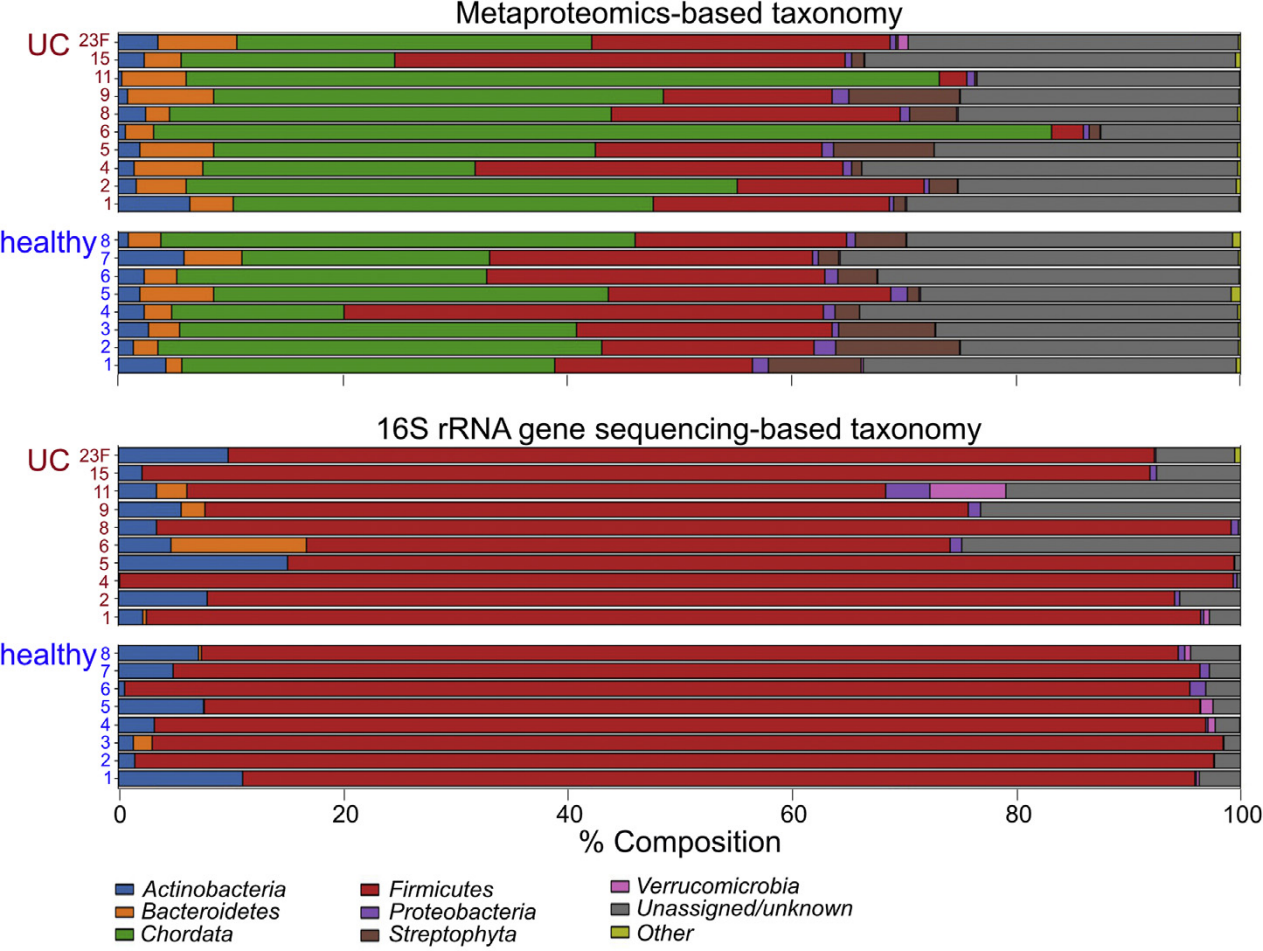
**Relative abundance plots for all 18 patient samples.** Comparison of microbiome phylum-level taxonomy by 16S rRNA gene amplicon sequencing (*lowe*r 18 bars; based on sequence counts) against LC-MS/MS bottom-up proteomics (*upper* 18 bars; based on peptide intensities, note that shared peptides were grouped in the “Unassigned/unknown” category). LC-MS/MS, liquid chromatography tandem mass spectrometry.

Large discrepancies in taxonomic resolution begin to emerge at the phylum level. By LC-MS/MS proteomics, we identified 46 phyla, including Ascomycota, Basidiomycota, Spirochaetes, Chordata, and Streptophyta in addition to the eight identified by 16S rRNA gene sequencing alone (Euryarchaeota, Actinobacteria, Bacteroidetes, Cyanobacteria, Firmicutes, Fusobacteria, Proteobacteria, and Verrucomicrobia) (Fig. 1). Firmicutes account for only 22.7% of the sample composition according to the LC-MS/MS; however, this phylum dominates the composition of patient microbiota according to 16S amplicon sequencing and accounts for 86.2% of all reads. Interestingly, the abundance of Bacteroidetes was relatively minimal by 16S rRNA gene sequencing (except for samples H5, UC9, UC15, UC23) yielding a Bacteroidetes:Firmicutes ratio of approximately 1:100 (Fig. 1). In contrast, Bacteroidetes account for an average of 4.2% of the microbiota peptide content by LC-MS/MS-based proteomics yielding a Bacteroidetes:Firmicutes ratio of approximately 1:5. The majority of identified peptides identified *via* metaproteomics predominantly originate from the Chordata phylum and are presumably host-derived. Additionally, a significant number of peptides are derived from the Streptophyta and are attributable to a variety of dietary plants, including *Solanum tubersum* (potato), *Seasum indicum* (sesame), *Theobroma cacao* (chocolate), *Zea mays* (corn), and *Oryza sativa* (rice) among many others [see Additional File 2]. Where comparable, we posit that differences in DNA extraction efficiencies (*e.g.*, Gram^+^ *versus*. Gram^-^), differences in metabolic/secretory activities, and shared tryptic peptides between microbes likely contribute to the discrepancy in taxonomic compositions between 16S gene sequencing and proteomics methods.

The trend of increased taxonomic diversity across microbiota samples observed by LC-MS/MS proteomics over 16S amplicon sequencing is conserved at each classification tier [Additional File 1, supplemental Figs. S1–S7]. At the species level, we detected a total of 848 species/strains by LC-MS/MS and only 38 by amplicon sequencing. Despite the predicted increase in diversity by LC-MS/MS, an average of 85.9% of the identifiable peptides are not mappable to a particular species. This observation is due to the redundancy and conservation of microbial proteins across distinct and divergent species. Thus, taxonomic predictions based on the peptide composition in samples become increasingly difficult with more granular classification levels.

### 176 Protein Groups Are Significantly Altered Between the Healthy and UC Cohorts

**Fig. 2:**
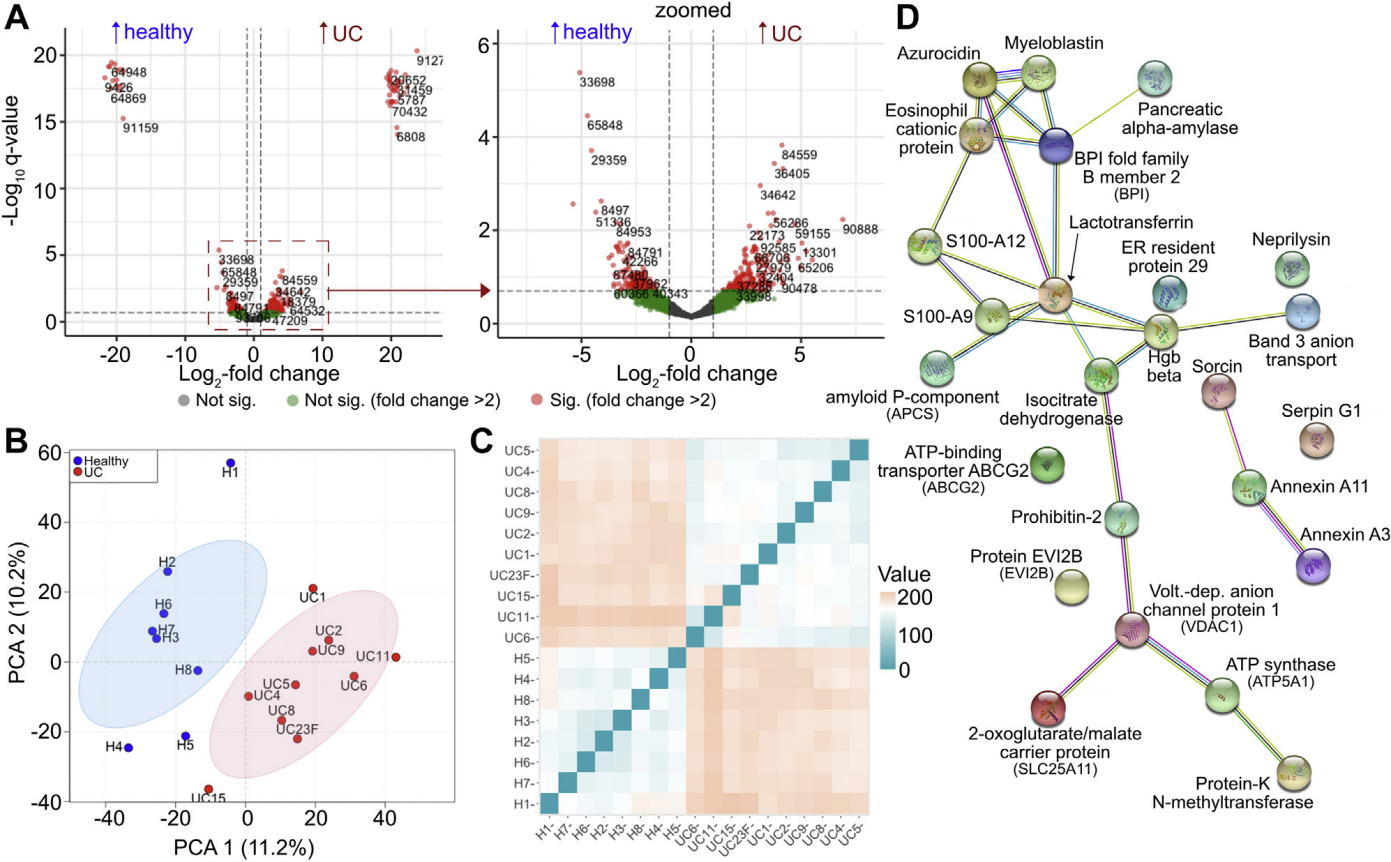
**Differential expression and protein network analysis of host and microbial proteins.***A*, volcano plot depicting differentially detected protein groups from patient fecal samples; whole plot (*left*), zoomed-in plot (*right*), *red* = significant (*q* < 0.1, foldchange > 2), *green* = not significant (*q* > 0.1, foldchange > 2), *gray* = N.S. (*q* > 0.1, foldchange ≤ 2), number adjacent red points represent unique “ClusterID” values corresponding to Additional File 4 labels. *B*, principal component analysis of all 18 patient samples across all differentially tested contrasts; *red* = healthy individuals, *blue* = UC patients. *C*, euclidean distance plot of all 18 patient samples across all differentially tested contrasts, *blue* = more similarity, *orange*/*red* = less similarity. *D*, STRING protein network analysis of host protein groups differentially enriched in UC patients’ fecal samples at medium confidence (0.400); edges: known interactions (*aqua*: from curated database, *magenta*: experimentally determined), predicted interactions (*green*: gene neighborhood, *red*: gene fusions, *blue*: gene co-occurrence), others (*chartreuse*: text mining, *black*: coexpression, *lavender*: protein homology). UC, ulcerative colitis

Differential expression analysis yielded 176 protein groups (from host, microbes, and diet) significantly altered between healthy and UC patients (*p* ≤ 0.005 and *q* < 0.1), with 65 groups enriched in healthy volunteers and 111 protein groups enriched in the UC volunteers (Fig. 2*A* and Additional File 3). Principal components analysis (PCA) of the dataset revealed a modest but distinguishable separation between the proteomic composition of healthy and UC fecal samples (Fig. 2*B*), which is in agreement with the Euclidean distance matrix generated for the same dataset (Fig. 2*C*).

Twenty nine of the 111 protein groups significantly enriched in UC fecal samples were host-derived; however, no host protein groups were enriched (*q*-value < 0.1) in the healthy individuals. STRING analysis of significantly enriched UC host proteins yielded 26 edges among 25 nodes and a highly significant protein–protein interaction (PPI) enrichment *p*-value of 1.84 × 10^−12^ at medium confidence (0.400) suggesting a strong association between tested proteins (Fig. 2*D* and Additional File 4) (72). At highest confidence (0.900), 13 edges between 25 nodes were found reinforcing a significant PPI enrichment *p* value of 2.0 × 10^−11^. At medium confidence, the most significant reactome pathways (fdr <0.001) were neutrophil degranulation, innate immune system, antimicrobial peptides, immune system, and metal sequestration by antimicrobial proteins. GO biological process terms (fdr <3.3 × 10−^8^; regulated exocytosis, neutrophil degranulation, secretion by cell, transport, leukocyte mediated immunity, and antimicrobial humoral response) and GO cellular component terms (fdr < 2 × 10^−7^; cytoplasmic vesicle lumen, secretory granule, secretory granule lumen, vesicle, cytoplasmic vesicle part, and cytoplasmic vesicle) associated with host proteins all support the assertion that host immune-related secretory events are prevalent in the gastrointestinal tracts of UC patients. Interestingly, because no host proteins were significantly enriched across healthy fecal samples, we posit that host-centric biological pathways associated with colitis occur in addition to rather than in lieu of processes associated with homeostasis.

More than half of the nonhost protein groups have limited to no annotations despite being significantly altered. For example, 37 of 65 and 48 of 82 nonhost protein groups enriched in the healthy and UC cohorts, respectively (*p* ≤ 0.005 and *q* < 0.1), were poorly annotated in the ComPIL database (*i.e.*, no annotation, annotated as hypothetical proteins or as domain of unknown function-containing proteins). While additional BLAST homology searches and InterProScan analyses were performed on each poorly annotated protein group, we were unable to make significant additional annotations for 11 of 37 protein groups enriched in the healthy cohort and 15 of 48 protein groups enriched in the ulcerative colitis cohort [see Additional File 4] (62, 63, 73). Such protein groups represent interesting targets for structural and biochemical validation as further inquiry could elucidate their possible roles, in the propagation of inflammatory or anti-inflammatory processes.

Significantly enriched and annotated nonhost protein groups among both healthy and UC cohorts had predicted and/or biochemically verified functions ranging from metabolism (glyceraldehyde-3-phosphate dehydrogenase and translation elongation factor Tu) to defense (type II secretion system protein). Notable nonhost entries that were increased in healthy volunteers include an acid-soluble spore protein (WP_071120403.1), methylene tetrahydrofolate reductase (SRS064276.159392-T1-C), and fruit bromelain (BROM1_ANACO). The enriched small spore protein is the only entry in its protein group and originates from the recently described bacterium *Romboutsia timonensis*, whose depletion has been associated with colorectal cancer incidence (74, 75). The methylene tetrahydrofolate reductase protein group enriched in the healthy cohort contains 29 members possessing similarity scores in the range of 98.6 to 100%. By BLAST analysis, these reductases likely originate from the Lachnospiraceae family of bacteria, and their examination could provide a glimpse into the microbial B-vitamin economy that importantly underpins host homeostasis, as humans are unable to *de novo* synthesize many essential B vitamins (76, 77, 78). Interestingly, fruit bromelain detected in four of eight healthy patient fecal extracts is a pineapple-derived cysteine protease we did not expect to encounter (79, 80). This protease is commonly sold as an over-the-counter supplement or as a component of meat tenderizers, and its detection may be an artifact introduced through patients’ diets.

With respect to protein groups enriched in UC patients, notable annotated nonhost entries include hyaluronan glucosaminidase (CLONEX_02,131), a transglycosylase SLT domain-containing protein (HMPREF0462_0704), and a metallohydrolase (WP_081140786.1). The enriched hyaluronan glucosaminidase group contains four members with almost identical sequences (99.95–100% identity). These proteins likely originate from the Lachnospiraceae family members *Tyzzerlla* or *Coprococcus*. Hyaluronan is a high-molecular-weight carbohydrate component of the human extracellular matrix that can serve as an inflammatory/injury signal for host immune receptors when degraded by host hyaluronidases (81). Hyaluronan glucosaminidase activity may exacerbate host inflammatory processes and contribute to UC-related inflammation, as well as afford microbes the ability to infiltrate host barriers. The transglycosylase SLT (lytic) domain-containing protein group includes 70 members with sequence identities of 95.71 to 100% compared with the *Helicobacter pylori* (*H. pylori*) enzyme. These enzymes catalyze the nonhydrolytic intramolecular cyclization of *N*-acetylmuramyl residues on bacterial cell walls propagating the cell wall remodeling process (82, 83). For *H. pylori*, previous studies demonstrate the importance of transglycosylase-generated cell wall muropeptide fragments to inducing host inflammation, which can in turn promote gut colonization (84, 85). Finally, the enriched metallohydrolase originates from *Pantoea latae* and is annotated as a nonpeptide amide C–N bond hydrolase that may inactivate amide-containing molecules such as lactams, which are contained in an important class of antibiotics (86, 87). A complete list of protein groups found differentially expressed in this patient cohort can be found in the supplement [see Additional File 4].

### Serine-type Endopeptidase Activity is Significantly Enriched in UC Samples

**Fig. 3:**
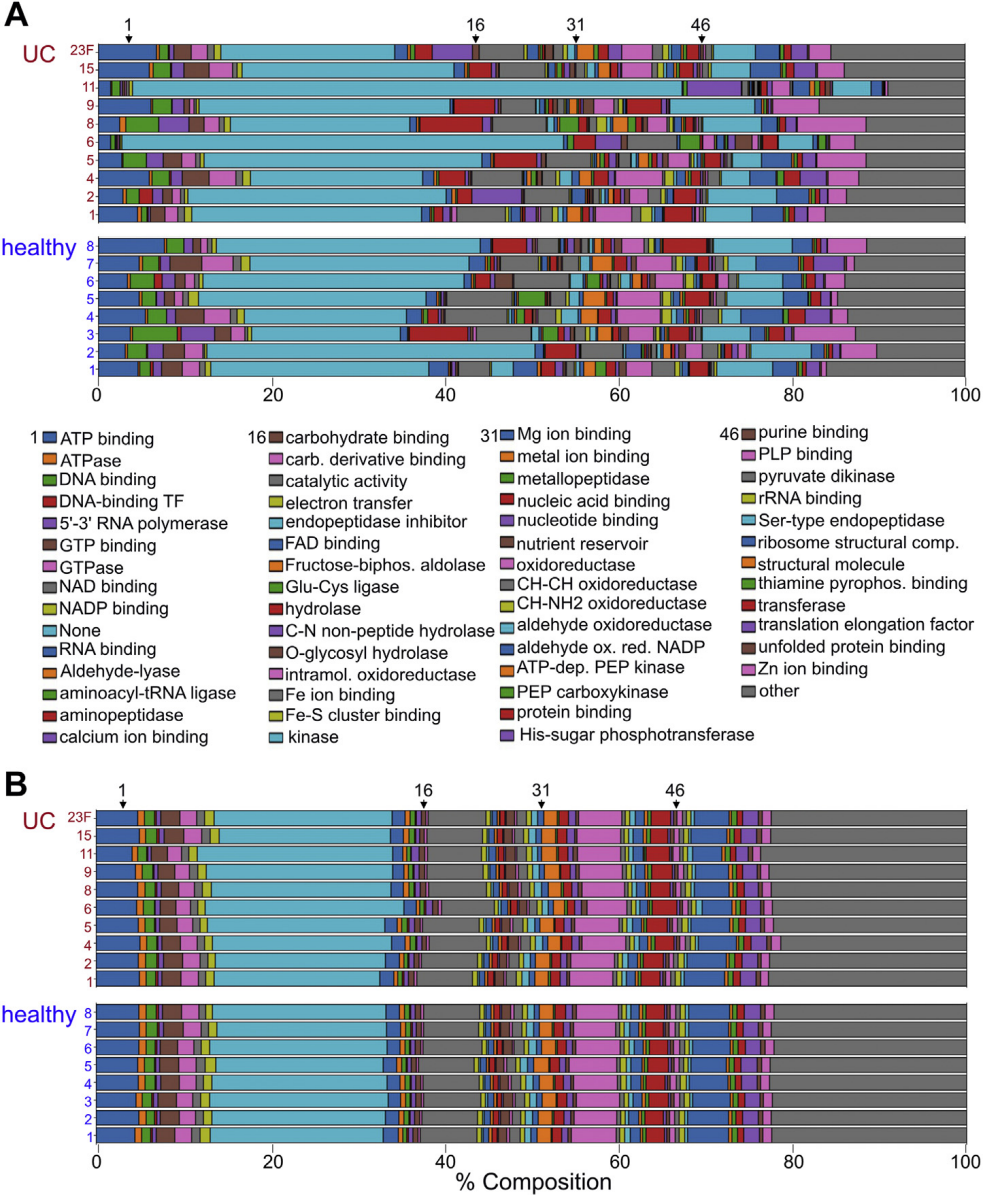
**GO molecular function relative abundance plots for all 18 patient samples**. Comparison of microbiota GO molecular function breakdown by LC-MS/MS using either (*A*) weighted measures (*upper* 18 bars; peptide intensity-based; to control for protein length, each GO term’s constituent protein group/cluster contributes the mean intensity of its constituent peptides, see Additional File 1 for more detail) or (*B*) unweighted measures (*lower* 18 bars; count-based; each GO term’s constituent protein group/cluster contributes one count). Bar segments represent the proportion of each GO term’s intensity or count relative to the total intensity or count (respectively) for each patient sample. Loosely, (*A*) represents GO terms as a function of protein copy number and (*B*) represents GO terms as a function of protein sequence diversity. GO, gene ontology; LC-MS/MS, liquid chromatography tandem mass spectrometry.

For GO term relative abundance analysis, we used mean peptide intensities for peptides within the same protein group to account for comparisons between proteins of different lengths. Of the 8538 quantifiable protein groups selected for relative abundance plotting, we identified 575, 394, and 85 terms for the molecular function, biological process, and cellular component GO namespaces, respectively. In general, GO relative abundance breakdowns between all samples for each namespace appear similar by unweighted (count-based) assembly (Fig. 3*B* and Additional File 1, supplemenal Figs. S8–S10). However, when weighted by corresponding ion intensities, GO term relative abundances between samples differ dramatically (Fig. 3*A* and Additional File 1, supplemental Figs. S8–S10). The “None” and “Other” categories occupy the largest areas both by unweighted and weighted assembly for all three GO namespaces. With respect to molecular function, global relative abundances (when averaged over all samples) for the terms glutamate-cysteine ligase activity (GO: 0004357), aminopeptidase activity (GO: 0004177), and serine-type endopeptidase activity (GO: 0004252) expand 31-, 18-, and 14-fold respectively going from an unweighted to weighted assembly. Conversely, global relative abundance for the terms enoyl-[acyl-carrier-protein] reductase (NADH) activity (GO: 0004318) and mismatched DNA binding (GO: 0030983) contract >50-fold going from unweighted to weighted assembly. Similar relative abundance expansions and contractions going from unweighted to weighted assemblies were observed for the biological process and cellular component namespaces [see Additional File 1, supplemental Figs. S9 and S10].

The large 31-fold unweighted-to-weighted relative abundance expansion of glutamate-cysteine ligase activity could be attributed to one protein group with one member (WP_027345637.1). This enzyme originates from *Hamadaea tsunoensis* and catalyzes a key step in the synthesis of glutathione, a key antioxidant for the microbiota (88). The 18-fold unweighted-to-weighted relative abundance expansion observed for the aminopeptidase activity term originated from 19 protein groups with one very dominant protein group (WP_027209280.1) representing 96.2% of the GO term namespace’s relative abundance. This predicted M18 family protease originates from *Butyrivibrio hungatei* but currently has no structural or biochemical annotation. Finally, the 14-fold expansion observed for the serine-type endopeptidase activity GO term is attributable to 38 protein groups with the majority share originating from host (85.7%) and minor shares originating from pig (13.5%) and microbes (0.8%). The serine-type endopeptidase from pig is an artifact, as it originates from the sequencing grade porcine trypsin used to generate peptides for LC-MS/MS analysis. Interestingly, human chymotrypsin-like elastase 3A (CEL3A_Human) and chymotrypsin-C (CTRC_human) are more abundant than porcine trypsin, resulting in 35.8% and 14.9% of the GO term share compared with porcine trypsin. Other prominent protein groups (less abundant than porcine trypsin) include chymotrypsin-like elastase 3B (CEL3B_human), cathepsin G (CATG_human), and trypsin-1 (TRY1_human) and comprise 11.3%, 5.1%, and 3.0% of the serine-type endopeptidase activity GO term, respectively.

We performed GO enrichment analysis in the R GOstats package with GO terms corresponding to differentially expressed protein groups serving as the enriched GO term set and GO terms mapping to all nondifferentially expressed protein groups as the “universe” set (64). GO terms overrepresented in either healthy or UC cohorts for each GO namespace (*p* < 0.01) are listed in the supplement [see Additional File 1, supplemental Figs. S11–S18].

For the healthy patient cohort, nine molecular function GO terms were enriched including hydrolase activity (hydrolyzing O-glycosyl compounds) (GO:0016787), cysteine-type peptidase activity (GO:0008234), rRNA binding (GO:0019843), and oxidoreductase activity (acting on iron–sulfur proteins as donors) (GO:0016730) among the most significantly enriched (*p* < 0.001, odds ratio >10). The biological process namespace contained nine GO terms enriched in healthy patients with polysaccharide catabolic process (GO:0000272), homeostatic process (GO:0042592), sulfur amino acid metabolic process (GO:0000096), nitrogen fixation (GO:0009399), and asexual sporulation (GO:0030436) among the most significantly enriched terms (*p* < 0.001, odds ratio >20). Only three GO cellular component terms were found to be enriched in healthy patients including oxidoreductase complex (GO:1990204), vanadium–iron nitrogenase complex (GO:0016613), and endospore-forming forespore (GO:0042601) (*p* < 0.001, odds ratio >100). Together the three GO namespaces strongly associate carbohydrate processing and microbial sporulation activities with healthy patients.

For the ulcerative colitis cohort, 21 terms were enriched in the molecular function GO namespace including calcium ion binding (GO:0005509), peptidase activity (acting on L-amino acid peptides) (GO:0008233), lipid binding (GO:0008289), catalytic activity (acting on a protein) (GO:0140096), serine-type endopeptidase activity (GO:0004252), serine hydrolase activity (GO:0017171), and calcium-dependent phospholipid binding (GO:0005544) (*p* < 0.001, odds ratio >10) [see Additional File 5]. The biological process namespace contains 18 entries including proteolysis (GO:0006508), aromatic amino acid family metabolic processes (GO:0009072), propionate metabolic process (methylcitrate cycle) (GO:0019679), acetate metabolic process (GO:0006083), short-chain fatty acid metabolic process (GO:0046459), antibacterial humoral response (GO:0019731), antifungal humoral response (GO:0019732), regulation of cytokine production (GO:0001817), and response to fungus (GO:0009620) among the most enriched terms (*p* < 0.003, odds ratio >10). The enriched cellular component term list was much shorter with only five entries, including extracellular space (GO:0005615), organelle lumen (GO:0043233), endoplasmic reticulum lumen (GO:0005788), intracellular membrane-bound organelle (GO:0043231), and extracellular region (GO:0005576) (*p* < 0.008, odds ratio >10). Together, these enriched GO terms associate the extracellular space/secretome, antimicrobial host responses and serine protease activity with UC patients in our study [see Additional File 1, supplemental Figs. S11 and S17]. Interestingly, serine proteases, and more broadly serine hydrolases, are an intensely studied class of enzymes with exquisitely selective mechanism-based fluorophosphonate probes described in the literature (89). These probes present an opportunity to better examine this class of enzyme in the context of human UC.

### ABPP Confirms the Presence of Active Serine-type Endopeptidases and Identifies Previously Undetected Serine-type Endopeptidases

**Fig. 4:**
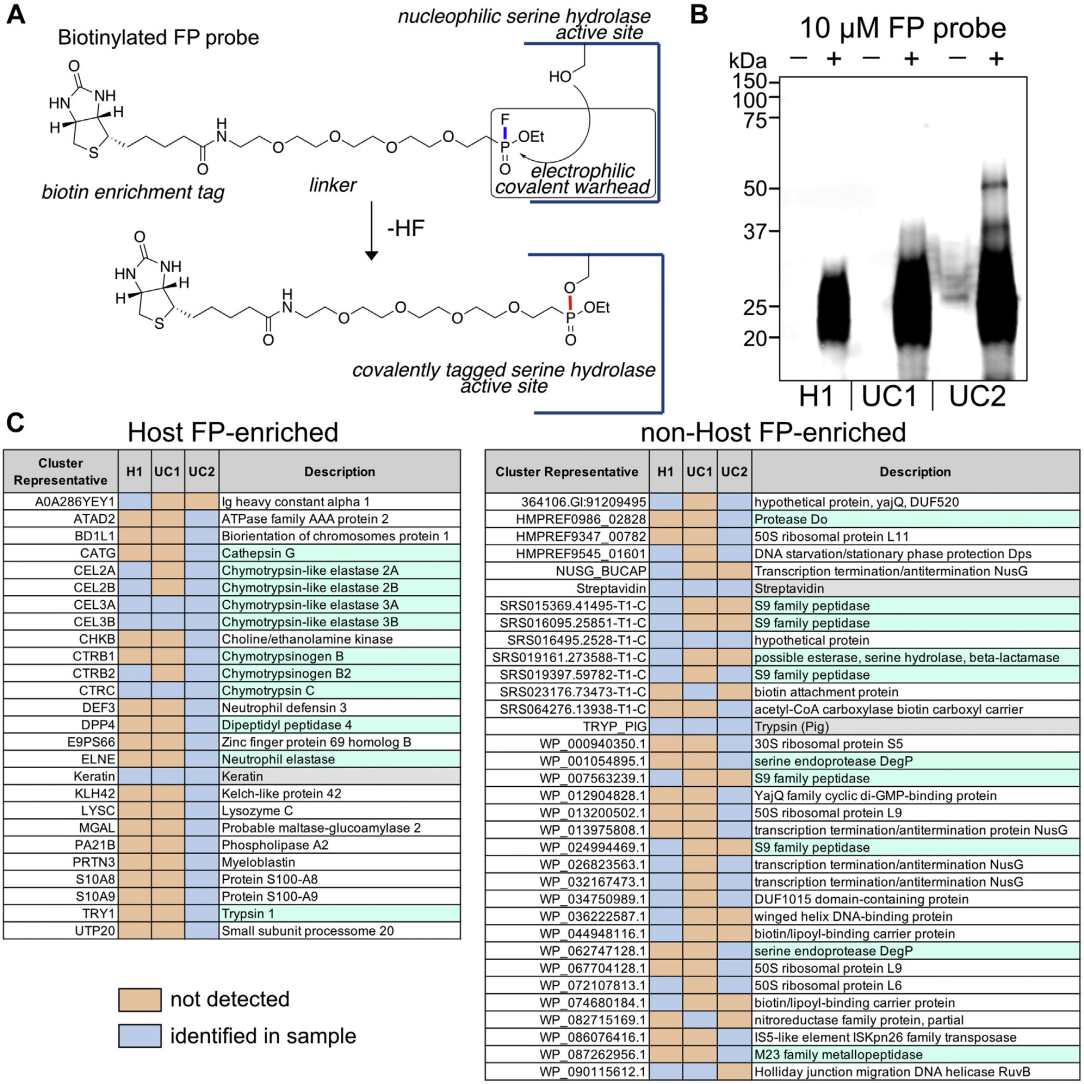
**Targeting serine hydrolases *via* activity-based protein profiling.***A*, FP probe structure and reaction schematic for covalent attachment to nucleophilic active-site serine in hydrolases. *B*, Western blot of three patient fecal lysates treated and enriched with FP probe followed by streptavidin bead enrichment visualized with fluorophore-conjugated streptavidin. Host (*C*) and nonhost (*D*) proteins from patient fecal samples enriched by FP probe and detected by LC-MS/MS (*blue* = protein detected in corresponding patient sample, *orange* = protein not detected in corresponding patient sample, *green highlights* = annotated proteases). FP, fluorophosphonate; LC-MS/MS, liquid chromatography tandem mass spectrometry.

We treated three patient fecal samples with a biotinylated fluorophosphonate-based (FP-based) serine-reactive probe to label and thus establish whether serine hydrolases were active in patient fecal samples (Fig. 4*A*) (89). Biotinylated fluorophosphonate probe (FP probe)-labeled proteins were enriched with streptavidin-agarose beads, visualized by Western blot (Fig. 4*B*) and identified by LC-MS/MS analysis using a previously described two-step large-to-focused database search strategy (90). Analysis of the LC-MS/MS data revealed several hundred noncontaminant protein sequences. These sequences were clustered into 104 distinct protein groups (95% similarity cutoff using CD-HIT) and further reduced to 63 protein groups with highly homologous host proteins condensed together. Of note, 27 and 35 protein groups derived from host (Fig. 4*C*) and nonhost (Fig. 4*D*) were identified, respectively.

The majority of host-derived protein groups labeled and enriched with the FP probe were also identified within the unenriched LC-MS/MS datasets [see Additional File 4]. Fourteen of 27 FP probe-enriched host proteins are known serine hydrolases including the chymotrypsin-like elastase family (2A, 2B, 3A, 3B), cathepsin G, dipeptidyl peptidase 4, neutrophil elastase, myeloblastin, trypsin 1, and phospholipase A2 (Fig. 4*C*). Enrichment of these particular hydrolases over all other host proteins provides confidence that the FP probe is selective for nucleophilic serine hydrolases in the tremendously complex fecal protein matrix. Aside from demonstrating that the hydrolases are active, these results also suggest that this fraction of proteases remain uninhibited by antiproteolytic proteins often found in the gut (91, 92, 93).

Most nonhost proteins are microbial in origin with the exception of streptavidin and porcine trypsin introduced during sample preparation (Fig. 4*D*). The most promising FP probe-susceptible proteins include protease Do entries (DegP), S9 family peptidases, and a beta-lactamase, as determined by sequence analysis. Interestingly, of the ten identified nonhost serine hydrolase protein groups, only one (SRS019397.59782-T1-C) was detected without FP-probe demonstrating the utility of chemical-based enrichment strategies for the identification of novel proteins in a complex environment. Of the 167,554 MS2 spectra collected for all FP-enriched LC-MS/MS data sets, only 5352 (3.2%) were assigned by ComPIL database searches. There is a strong likelihood that other serine hydrolase-derived peptides are present in our microbiota samples, but they remain unidentified due to limitations imposed by the incompleteness problem associated with metaproteomics database searching (26). Unfortunately, the techniques for database-independent, high-confidence identification of these peptides and their parent protein sequences are currently not well established.

### *De Novo* Peptide Sequencing Enables Glimpses into the Dark Peptidome

Of the 2,829,920 MS2 fragmentation spectra we collected overall, 523,155 (18.5%) were matched to a corresponding peptide with a 1% peptide false positive rate using a target-decoy search strategy paired with the ComPIL database. The modest number of peptide spectrum matches we observed is likely attributable to [1] a loss in filtering sensitivity that often accompanies database expansion (90, 94) and [2] a never-complete database that is perennially associated with metaproteomics. We posit that a nontrivial portion of unmatched MS2 spectra map to either known or unknown peptide sequences, and we aim to estimate the size of unmatched MS2 spectrum space or “the dark peptidome,” using a complimentary *de novo* peptide sequencing approach (95).

**Fig. 5:**
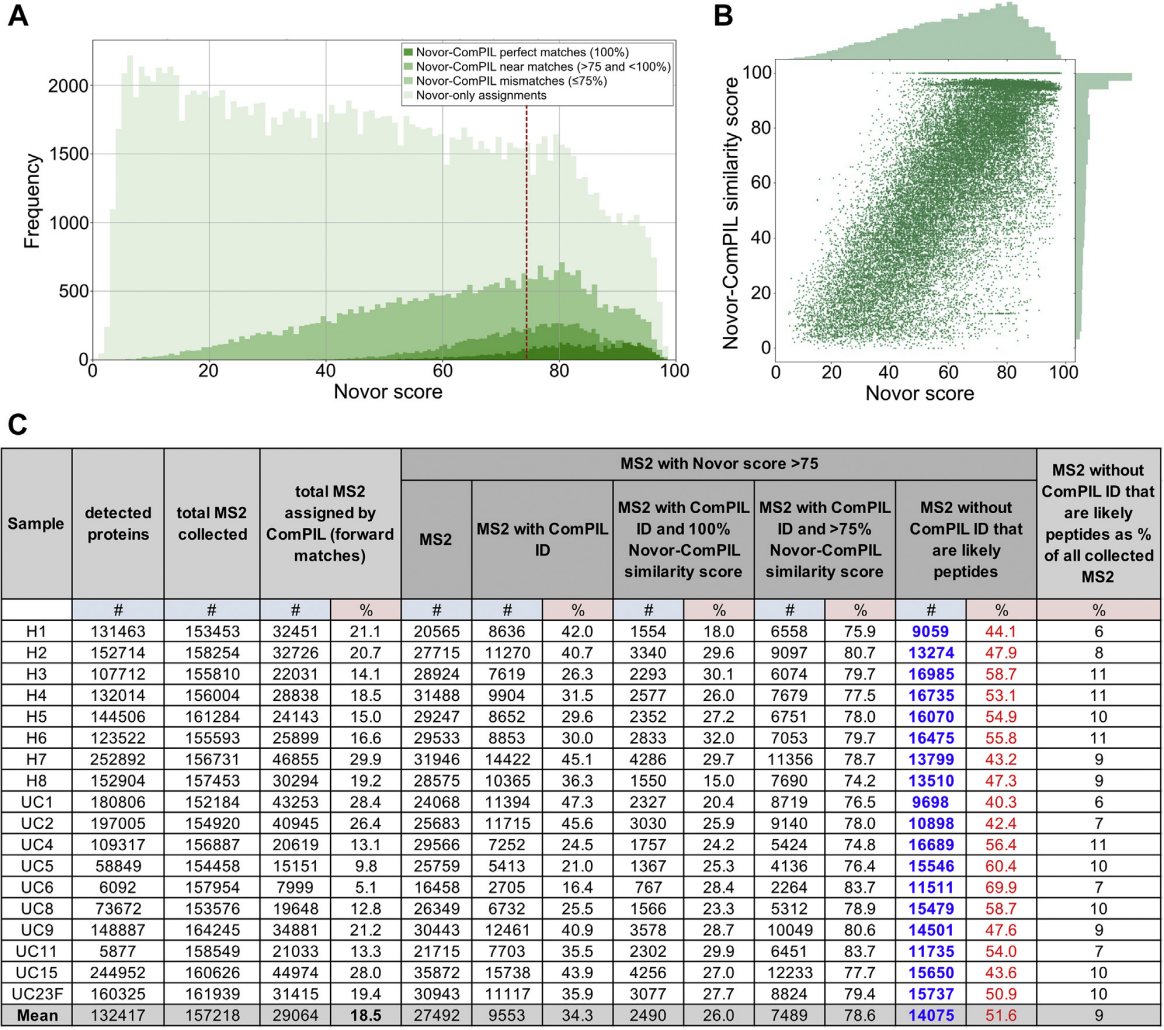
**Estimating the size of database-elusive peptide (“dark peptidome”) space *via de novo* peptide sequencing.***A*, overlapping histograms of all H2 patient sample MS2 spectra by Novor score (0–100, x-axis); *darkest green area* represents MS2 correctly assigned by Novor determined by comparison to ComPIL (database) result; *lightest green area* represents MS2 without ComPIL peptide assignments; *dashed vertical red line* represents Novor score = 75 cutoff and MS2 to the *right* of this line were used to estimate the size of unassigned peptide space. *B*, joint plot of H2 patient sample MS2 spectra depicting correlation between Novor score (0–100) and Novor-ComPIL similarity score (0–100). *C*, number of MS2 spectra from all patient samples with Novor scores >75 that likely represent peptides but do not have ComPIL peptide matches (“dark peptidome”). With duplicates removed, the total number of proteins detected between all 18 samples is 576,625. ComPIL, comprehensive protein identification library.

We subjected MS2 spectra from all patient fecal sample LC-MS/MS datasets to *de novo* peptide sequencing using the Novor algorithm (96). Novor attempts to deduce peptide sequence from MS2 fragmentation spectra, generating a *de novo* peptide spectrum match (PSM) and an accompanying confidence score (Novor score; higher scores indicated better predicted matches). Where available, we paired *de novo* PSMs with their corresponding database PSMs (ComPIL2-assigned) and calculated an additional Novor-ComPIL2 similarity score (*de novo* database similarity score) based on the Needleman–Wunsch comparison algorithm (raw scores were scaled to 100, where 100 represents a perfect match) (68). We used these values to construct overlapping histograms [Fig. 5*A* and Additional File 1, supplemental Figs. S19 and S20] and joint plots [Fig. 5*B* and Additional File 1, supplemental Figs. S21 and S22] depicting possible unidentified peptide space in patient fecal microbiota samples.

ComPIL database-assigned PSMs were nonuniformly distributed along the Novor score axis with a larger proportion of database PSMs grouped near the high-confidence *de novo* sequencing Novor scores (69, 70, 71, 72, 73, 74, 75, 76, 77, 78, 79, 80, 81, 82, 83, 84, 85, 86, 87, 88, 89, 90, 91, 92, 93, 94, 95, 96, 97, 98, 99) (Fig. 5*A*). While perfect *de novo* database agreements were rare, a large proportion of database PSMs possessed strong similarity to *de novo* PSMs, a relationship best depicted by a Novor score *versus* Novor-ComPIL2 similarity score joint plot (Fig. 5*B*). Thus, it is reasonable to expect that above a conservative cutoff value (Novor score = 75), significant numbers of MS2 spectra without database assignments correspond to peptides that either are not contained in the ComPIL2 database or were rejected due to our high search-filter stringency. Based on this assessment, it is estimated that an average of 14,075 MS2 spectra with a Novor score of 75 or greater remain unidentified per sample (Fig. 5*C*). This corresponds to approximately 9% of all MS2 spectra per patient sample, which could increase global identifications by approximately 50%. Note that 9% is a lower limit estimate for global unidentified MS2 spectra as this value is based only on MS2 spectra with Novor scores >75. A significant fraction of MS2 with Novor scores <75 have been identified by ComPIL and further suggest that the upper limit of unidentified MS2 is much larger than 9% (Fig. 5*A*, left of red vertical dashed line). BLAST analysis of several *de novo* PSMs, which do not have corresponding database PSMs, returned reasonable, high-similarity score matches to microbial peptides from the NCBI nonredundant database supporting this assertion. Below a Novor score of 75, there are likely many unidentified peptide-MS2; however, these peptides are also likely intermixed with many non-peptide-MS2.

## Discussion

16S rRNA gene amplicon sequencing has been a workhorse technique for microbiota studies over the last decade due in large part to the simplicity of material extraction and the broad availability of resources needed to generate meaningful data. In contrast, the use of LC-MS/MS-based metaproteomics profiling has been more sparse for the opposite reasons. While functional proteomic interrogation of the microbiome by LC-MS/MS was our main goal, we considered it important to contrast our proteomics-based taxonomy findings with those generated by 16S sequencing, a technique more familiar to the microbiome research community. Gut microbiome taxonomy through the lens of 16S gene analysis *versus* LC-MS/MS-based proteomics is expectedly different, but in some unexpected ways (34). At the phylum level, we expected to see a similar configuration by both techniques with the exception that proteomics would include a small concession for host proteins. Unexpectedly, we observed both host and diet-derived (potato, rice, corn, *etc.*) proteins in great relative abundance to microbial proteins. Our analyzed samples were pre-enriched for microbes by filtration, differential centrifugation, and several washing steps, yet nearly half of the detected proteome was mapped back to host or dietary plant proteins. Another unexpected finding was a discrepancy between the relative abundance ratios of Bacteroidetes and Firmicutes. This discrepancy could be artifactual and originate from differences in DNA extraction efficiencies between microbes, but it could also be the result of biologically relevant phenomena. For example, overrepresentation of Firmicutes by 16S sequencing could stem from an abundance of Firmicutes cells that are metabolically inactive (spores) relative to Bacteroidetes cells (97). This would be in agreement with our finding that the asexual sporulation GO term is enriched in healthy patient samples. Finally, we found that at more granular taxonomic strata [see Additional File 1, supplemental Figs. S1–S7], the number of identifiable organisms was unexpectedly greater for proteomics (using unique peptides as a proxy) than for 16S sequencing. By proteomics, we observed peptides originating from hundreds of organisms at the species level *versus* several dozen by 16S. Note that in our case, a standard 16S V4 analysis by paired-end short-read sequencing was performed; however, other higher-resolution techniques are becoming more accessible (98, 99, 100). Expectedly, the proportion of uniquely mappable peptides progressively decreased at more granular taxonomic levels such that at the species level, about 75% of all peptide intensity could not be mapped to a particular species. Because peptides are proxies for both taxonomy and function, this observation hints at a functional redundancy among microbes in the gut, which could be better examined by differential expression and GO term analysis of proteomics data.

One of the leading motivations for performing differential expression analyses on microbiome samples is to identify specific biomarkers or disease-associated microbial proteins for further examination. Toward this goal, we identified 176 protein groups significantly enriched (*q* < 0.1) in either healthy or UC volunteers, with a major share originating from microbes. Interestingly, no host proteins were identified as significantly enriched in the fecal extracts of healthy volunteers while several were found enriched in the fecal extracts of UC patients. Among the host proteins enriched in UC patients, we identified the calibrating entry, protein S100-A9 (*p* < 0.004, *q* < 0.07), a component of fecal calprotectin and established IBD biomarker (101, 102). According to STRING and reactome analyses, many host proteins enriched in UC patients are also inflammation-aligned lending more credibility to the prospect that the enriched proteins we have identified are truly UC-associated. For a comprehensive list of enriched protein groups, see Additional File 4. While most enriched protein groups had some annotation, a significant portion had little to none. This finding presents an exciting opportunity for the structural and biochemical study of novel sequences. Given the enormous number of domains of unknown function (DUF) and unknown function-type proteins catalogued from microbiome metagenomic sequencing efforts, we are faced with a prioritization problem wherein the most disease-relevant sequences are obscured by less impactful ones (103). LC-MS/MS-based proteomics appears in this context to be an important tool for identifying sequences that are both expressed and biologically relevant, which will help focus our future studies. Lastly, it is important to point out that poorly annotated proteins (*i.e.* proteins without GO assignments) factor weakly or not at all into broader functional analyses like GO enrichment simply due to the nature of enrichment testing (*i.e.* hypergeometric). Therefore, novel sequences without known biological- or disease-relevance are important to eventually characterize.

Within the detected microbiome proteome, known functional diversity is high with several hundred molecular function GO terms represented. A flat depiction of molecular function wherein a 1-sequence-1-count paradigm is applied reveals a consistent relative abundance configuration between all samples. We reasoned that count-based GO term depictions effectively reveal molecular diversity as each GO term’s constituent protein group/cluster equally contributes to a term’s size. However, this measure alone fails to capture material abundance. To depict material abundance, we have instead weighted GO terms by the peptide intensities (a very loose proxy for protein copy number) of their constituent protein groups/clusters (see Additional File 1 for details regarding this calculation procedure). Interestingly, when this paradigm is applied, a very different picture emerges. The relative abundance of many molecular function GO terms shifts, sometimes dramatically. One of the most conspicuous terms to us was “serine-type endopeptidase activity” that expanded an average of 14-fold among all patient samples going from count-based to intensity-based representation. Additionally, we found this same term enriched in UC patient fecal samples by hypergeometric testing, warranting a closer inspection of the protein groups that contribute to this term. We found that the major contributors were host-derived serine proteases (85.7%) such as chymotrypsin-C and the chymotrypsin-like elastase family with minor contributions from porcine trypsin (13.5%) (an artifact of sample preparation) and microbial serine proteases (0.8%). The high relative abundance of serine proteases in fecal samples is not surprising given that they are important components of host digestive enzyme cocktails secreted into the gut lumen. We were, however, surprised to find both host and microbial serpins, which are known active-site directed suicide inhibitors for serine/cysteine proteases, in fecal samples. This observation suggests that there might be important regulatory host–microbe cross talk with respect to proteolytic activity that occurs in the gut. By comparing the abundance of proteases or serpins, it would still be difficult to determine which and what fraction of serine proteases remained active upon fecal sample collection. To identify active serine proteases, we treated fecal samples with an active-site directed serine-hydrolase selective chemical probe (FP probe) for labeling, enrichment, and target identification *via* LC-MS/MS (ABPP) (29, 30, 89). We examined three patient fecal samples (one healthy, two UC) and qualitatively found human chymotrypsin-like elastases 3A and 3B and chymotrypsin-C enriched and therefore active in all samples. For one UC sample (UC2), we identified additional FP probe-enriched host proteases including cathepsin G, chymotrypsin-like elastase 2A and 2B, dipeptidyl peptidase 4, neutrophil elastase, and trypsin 1, lending support to the idea that aberrantly increased protease activity is associated with IBD (92, 104). In addition to host proteases, we identified several microbial proteases from all three patient samples upon FP probe enrichment. Surprisingly, we found that nine of ten microbial proteases were not detected by LC-MS/MS at all without FP probe enrichment. This finding suggests that there are likely many microbial proteases expressed in the gut microbiota and that they are likely below the limit of detection by most current sampling and LC-MS/MS profiling strategies. We speculate that this sentiment also holds true for other low-abundance, high-impact protein functionalities, underscoring the importance of pre-enrichment strategies for future proteomics studies.

In addition to sampling limits, many microbial peptides that are sampled by LC-MS/MS are liable to go undetected due in large part to database-completeness limitations. We attempted to estimate the number of peptide-likely fragmentation spectra per LC-MS/MS experiment using a *de novo* sequencing tool (Novor) in order to define a rough boundary around the amount of unassigned peptide space captured by the mass spectrometer but unidentified by our database workflow. Homology searching of high-confidence peptide-like fragmentation spectra revealed high numbers of exact and near-matches to peptide sequences in the NCBI nonredundant database. Though *de novo* peptide sequencing coupled to homology searching can help capture database-elusive peptides in more defined contexts (95), we were reluctant to rely more heavily on this strategy without a stringent methodology for distinguishing between sequencing errors and truly homologous peptides, especially in a context as taxonomically diverse as the gut microbiota. An additional layer of difficulty rests in determining how to treat *de novo* only peptides that are constituents of completely unknown parent protein sequences. Deep genome sequencing and the use of custom MAGs are an obvious path forward especially as long-read technologies become more accurate and accessible (15, 22, 23, 24, 25, 105, 106, 107, 108). Notably, long-read technologies are expected to yield more contiguous genome assemblies, thus accelerating the functional annotation process for novel sequences by enhancing our ability to contextualize these novel sequences within their respective genomes. For peptides/proteins that elude this approach, however (low abundance microbes, heavily posttranslationally modified peptides/proteins, nonribosomal peptides/proteins, *etc.*), perhaps genomics-agnostic, middle- and top-down proteomics sequencing could be applied (109, 110). We anticipate that the expanded use of ABPP techniques in the microbiota will enrich for many protein sequences not contained in large databases such as ComPIL, and robust high-throughput methods for identifying these whole novel protein sequences will be needed.

In summary, we identified 176 discrete host and microbial protein groups differentially enriched between healthy and UC patients. Our analysis revealed several protein functions associated with ulcerative colitis, with the function “serine-type endopeptidase activity” featuring prominently. We also identified host and microbial serine protease inhibitors in concert with serine proteases. Using an activity-based chemical tagging strategy, we were able to enrich for serine hydrolases/proteases and showed that these enzymes are still active in the gut despite the presence of active-site directed protease inhibitors. This strategy also revealed the presence of previously undetected serine proteases demonstrating the utility of activity-based tagging for the amplification of low-abundance proteins. Finally, we paired our database metaproteomics strategy with *de novo* peptide sequencing to estimate the size of high-confidence peptide space in our samples that remains unidentified despite the use of a large comprehensive database. Our data suggests that at the lower bound, at least an average of 9% of all our collected fragmentation spectra per run likely correspond to peptides, but remain unmatched.

## Data Availability

The datasets generated and analyzed during the current study are available in the Proteomics Identification Database (PRIDE) repository (69) under project PXD022433 or the Zenodo data repository (55) with https://doi.org/10.5281/zenodo.5717460.

## Supplemental data

This article contains supplemental data.

## Conflict of interest

The authors declare no competing interests.
